# Draft Genome Sequence of *Shewanella baltica* M1 Isolated from Brackish Surface Water of the Gulf of Gdańsk

**DOI:** 10.1128/genomeA.00611-16

**Published:** 2016-06-23

**Authors:** Joanna Karczewska-Golec, Dominik Strapagiel, Marta Sadowska, Agnieszka Szalewska-Pałasz, Piotr Golec

**Affiliations:** a Department of Molecular Biology, University of Gdańsk, Gdańsk, Poland; b Department of Molecular Biophysics, Faculty of Biology and Environmental Protection, Biobank Laboratory, University of Łódź, Łódź, Poland; c Laboratory of Molecular Biology (affiliated with the University of Gdańsk), Institute of Biochemistry and Biophysics, Polish Academy of Sciences, Gdańsk, Poland

## Abstract

Here, we present the 5.168-Mbp draft genome sequence of *Shewanella baltica* M1, the first *Shewanella* strain from the Gulf of Gdańsk to have its genome sequenced and annotated. The availability of the genome sequence of strain M1 will promote further global analyses of bacterial stress responses in the unique Gulf of Gdańsk ecosystem.

## GENOME ANNOUNCEMENT

*Shewanella baltica* M1 was isolated in summer 2005 from surface water of the Gulf of Gdańsk (54°33′01.06″N, 18°39′45.50″E), a southeastern bay of the Baltic Sea. Water in this unique basin is subject to substantial hydrological and hydrochemical variability, which results from large anthropogenic impact, dynamic interactions of marine and fresh water, and the morphometry of the Gulf of Gdańsk itself (1). For instance, while the average surface salinity of the Gulf of Gdańsk is about 7 PSU, it periodically drops to around 1 PSU in nearshore waters (2). These varied natural and anthropogenic impacts affect the functioning of the unique Gulf of Gdańsk ecosystem (3).

The genus *Shewanella* comprises a phenotypically diverse group of bacteria with a worldwide distribution (4). *S. baltica* strains occupy primarily aquatic and sedimentary niches that are chemically stratified on a permanent or seasonal basis (5). They were also shown to be the major contributors—among the H_2_S-producing species—to the spoilage of iced marine fish (6). To successfully compete in such varied environments and efficiently respond to the niche-specific resources, *S. baltica* has developed robust respiratory, metabolic, sensing, and regulatory systems (7). Here, we report the draft genome sequence of strain M1, the first *Shewanella* strain from the Gulf of Gdańsk to have its genome sequenced. The strain was identified based on matrix-assisted laser desorption ionization–time of flight mass spectrometry profiling, confirmed by the whole-genome sequencing reported here.

Whole-genomic DNA was purified with the use of the Sigma GenElute bacterial genomic DNA kit from a fresh overnight culture. Illumina paired-end libraries were prepared with 1 ng of genomic DNA using the Nextera XT kit. A total of 9,591,472 paired-end reads were generated on the Illumina NextSeq500 platform at a read length of 2 × 150 bp. Reads were analyzed and quality-checked using FastQC version 0.11.13 (http://www.bioinformatics.babraham.ac.uk/projects/fastqc). Low-quality data were filtered such that, for a pair of paired-end reads, each read had more than 90% of bases with a quality score greater than or equal to Q20. Genome assembly was performed using SPAdes version 3.7.1 (8), combing *de novo* assembly and manual editing. The final assembly consisted of 91 contigs (>500 bp) totaling 5,167,578 bp, with a GC content of 46.16 mol% and an average genome coverage of 34×. The *N*_50_ and *N*_75_ contig lengths were 143,280 bp and 76,941 bp, respectively.

The closest whole-genome sequences are those of *S. baltica* OS625 (85.84% symmetrical identity and 96.75% gapped identity) and *S. baltica* OS185 (84.94% symmetrical identity and 96.78% gapped identity) (9) among the draft and the complete genome sequences, respectively, in NCBI.

The draft genome of strain M1 was annotated using the Prokaryotic Genome Annotation Pipeline version 3.1 at NCBI (10). The annotation revealed 4,270 predicted protein-coding sequences, 92 tRNA genes, and 14 rRNA operons.

The availability of the genome sequence of *S. baltica* M1 will facilitate further transcriptome- and proteome-centric approaches to explain how versatile *Shewanella* strains functionally interact with their habitats.

### Nucleotide sequence accession numbers.

This whole-genome shotgun project has been deposited at DDBJ/EMBL/GenBank under the accession number LWED00000000. The version described in this paper is the first version, LWED01000000.
